# Scintigraphy for the diagnosis of primary unrecognised fractures in multiple trauma patients - a prospective, blinded, monocentric study

**DOI:** 10.1007/s00068-025-02865-z

**Published:** 2025-05-12

**Authors:** Arne Wilharm, Philipp Schenk, Kay Becker, Lina Van Nie, Joachim Hillmeier, Gunther Olaf Hofmann, Dominik Wilkens

**Affiliations:** 1 Department of Trauma, Hand and Reconstructive Surgery, University Hospital Jena, Am Klinikum 1, 07747 Jena, Germany; 2 Research Executive Department, BG Klinikum Bergmannstrost Halle gGmbH, Merseburger Strasse 165, 06120 Halle (Saale), Germany; 3 Department of Diagnostic and Interventional Radiology, St. Vincenz Hospital, Auf dem Schafsberg, 65549 Limburg, Germany; 4 Center for Orthopaedics and Traumatology, St. Vincenz Hospital, 65549 Auf dem Schafsberg, Limburg, Germany

**Keywords:** Multiple trauma, Scintigraphy, Missed injury, Trauma scan, Polytrauma

## Abstract

**Introduction:**

After structured (S3 guideline, ATLS^®^) acute care of multiple trauma patients in trauma centers, post-acute missed injuries continue to occur with incidence rates ranging from 1.3 to 39% as described in literature. The aim of the tertiary survey was the reduction of these rates. However, high numbers of missed injuries continue to be reported. The aim of this prospective, single-center, blinded clinical diagnostic study was to determine whether the standardised use of 3-phase whole-body skeletal scintigraphy in severely injured patients can reduce the number of missed injuries compared with the established standard procedure for polytrauma diagnosis.

**Methods:**

26 patients aged 18 years or older (median 53.5 years, 4 female, 22 male) with an ISS ≥ 9 were evaluated by an orthopaedic and trauma surgeon using skeletal scintigraphy after completion of standardised trauma room diagnostics and tertiary survey, a median of 7 days after trauma. All clinical and diagnostic examinations were then analysed and a final consensus was reached on the bony injuries. An evaluation of each procedure against the consensus was performed for the whole body and five body regions.

**Results:**

Skeletal scintigraphy was clearly superior to the established method (sensitivity 98.8% vs. 75.4%). Of the 60 additional bony injuries identified, 25 were treated without therapeutic consequences. Twenty-nine were treated conservatively without additional immobilisation and five with additional immobilisation. One unnecessary immobilisation was ended and no surgical treatment was required.

**Conclusion:**

Three-phase whole-body skeletal scintigraphy is a low-risk, highly sensitive tool for reducing the incidence of missed injuries. A more liberal indication for skeletal scintigraphy should be given for injuries of increasing severity and in persons with impaired consciousness or paralysis, to avoid sequelae of missed injuries.

**Clinical trial registration:**

The study was registered at the German Clinical Trails Register (DRKS) with the identifier DRKS00029402.

## Introduction

Delayed or overlooked injuries were referred to in the literature already in 1991 as the trauma surgeon’s “nemesis” [1]. Prior to the introduction of polytrauma spirals (whole body trauma scans), the rate of delayed diagnoses of injuries in seriously injured patients was between 1.3% and 39%, of which 15–22.3% were classified as clinically relevant. In their review, Pfeifer et al. summarise the results of 20 studies from 1980 to 2006 in which delayed diagnoses were identified by reviewing a patient’s records, re-examining the entire diagnostic process or re-examining the patient several months after the trauma [2]. In one registry study, among 26,264 patients who had been diagnosed by means of polytrauma spiral CT after trauma, 215 patients (0.82%) were identified in whom injuries were detected only after a delay [3]. Kok et al. reported in 2024 that despite polytrauma spiral CT, 13.9% of the 697 patients in the study had delayed diagnosis. This was particularly true for patients who were primarily admitted to intensive care or had an ISS ≥ 16. Half of the delayed injuries (49.5%) were imaged on CT [4].

To this day, despite polytrauma spiral CT and re-evaluation of patients and diagnostics as part of a tertiary survey according to ATLS, there are still overlooked injuries. However, the rate of undetected bony injuries in seriously injured patients is unclear, as the data in the literature are based only on follow-up examinations or registry evaluations and there are no studies in which the polytrauma spiral CT was compared to another highly sensitive method.

In 1992, Spitz et al. reported in a retrospective evaluation of 162 skeletal scintigraphies of severely injured patients that additional fractures were found in approximately ¼ of all cases. They were of the opinion that whole-body skeletal scintigraphy should be part of routine care for multi-trauma patients, similar to staging diagnostics for malignancies [5]. At the time, other authors also reported a high rate of additional fractures detected by skeletal scintigraphy in severely injured patients; for example, Runkel et al. 1993 found 68 additional fractures in 53 patients examined [6]. Despite high sensitivity and low radiation exposure for patients, skeletal scintigraphy has not become established as a routine examination for severely injured patients and has been forgotten for this indication.

### Study objectives

The aim of this monocenter, prospective and blinded study was to determine whether additional fractures in multiply and seriously injured patients (ISS ≥ 9) can be detected by means of skeletal scintigraphy and whether these have clinical relevance, despite a polytrauma spiral CT and ATLS-compliant triple clinical examination.

### Patients and methods

#### Study design

All adult (≥ 18 years), multiply or seriously injured patients (ISS ≥ 9) who were admitted to the emergency room of St. Vincenz Hospital Limburg, a regional TraumaZentrum DGU^®^, between 3 September 2019 and 18 June 2021 were included in the study with their consent (Table 1). The emergency room treatment was carried out in accordance with ATLS, including primary and secondary surveys, a polytrauma spiral CT and, if necessary, additional conventional X-rays. After four days, a standardised clinical examination by a specialist in the context of a tertiary survey was carried out to identify additional injuries. In the course of this follow-up, additional regions suspected of fracture were documented. On the sixth day after the trauma, a 3-phase whole body scintigraphy with Technetium Tc99m was performed. Based on the findings from the trauma room, the results of the follow-up clinical examination and the scintigraphy, further specific radiological diagnostics were then carried out.

**Table 1:** ; study inclusion and exclusion criteria

Inclusion Criteria	Exclusion Criteria
Age ≥ 18 years	Age < 18 years
Gender Male, Female, Diverse	
ISS ≥ 9 points and < 75 points	ISS < 9 points or = 75 points
Inpatient admission	No inpatient admission
Consent to the study	No consent for study
whole body CT, scintigraphy and tertiary survey performed	Patient transferred or discharged before scintigraphy or tertial survey

### Polytrauma spiral CT

All CT examinations were carried out using a Siemens computer tomograph (Somatom Definition Flash VA 48 A with Somaris 7 Syngo CT VA 48 A operating software). Non-contrast examination of the neurocranium (slice thickness of the reconstructions: 4 mm) was carried out first, followed by the non-contrast investigation of the cervical spine (slice thickness of the reconstructions: 2 mm). After intravenous administration of contrast agent (110 ml Imeron 350) with a delay of 80 s, the “whole body CT” was carried out from the skull base to the lesser trochanter minor, or in the case of injuries to the lower extremities, possibly including the feet (slice thickness of reconstructions: 2 mm). Arms were positioned on the body.

As part of this study, Analysis of the polytrauma spiral CT was carried out by a radiology specialist who was unaware of the clinical findings and the scintigraphy findings, as well as the radiological findings of the examination in the context of the actual clinical treatment.

### Skeletal scintigraphy

All skeletal scintigraphies were carried out using a double-head camera from General Electric (Discovery NM 630). In each case, a 3-phase scintigraphy with imaging of the entire body and an additional SPECT examination was carried out.

The skeletal scintigraphy was evaluated by a specialist in nuclear medicine as part of the study, without knowledge of the clinical findings and the findings of the polytrauma spiral CT, as well as the radiological findings of the examination in the context of the actual clinical treatment.

### Verification of additional scintigraphic findings

All fractures identified by scintigraphy were verified by new CT, with slice optimisation, additional CT scan or, in cases of doubt, MRI in order to rule out other causes for the enhancement.

### Data collection

The fractures identified in the three examinations (polytrauma spiral CT, tertiary survey with clinical examination, skeletal scintigraphy) were recorded in an Excel database created for this purpose (Microsoft, Redmondon, USA). A total of 170 possible fracture localisations were distinguished.

### Statistical analysis

Statistical analysis was performed using SPSS V.26 (IBM SPSS Statistics for Windows. Armonk, NY: IBM Corp.). In addition to descriptive statistics, the sensitivity, specificity, positive predictive value (PPV) and negative predictive value (NPV) were calculated. Furthermore, Phi and Kappa were calculated as measures of the correlation or agreement of the examination results (Table 1).

**Table 2:** Interpretation of phi coefficient and Cohens kappa

Phi Coefficient		Cohens Kappa	
+ 0.70 or higher	Very strong positive relationship	0	agreement equivalent to chance
+ 0.40 to + 0.69	Strong positive relationship	0.10–0.20	slight agreement
+ 0.30 to + 0.39	Moderate positive relationship	0.21–0.40	fair agreement
+ 0.20 to + 0.29	weak positive relationship	0.41–0.60	moderate agreement
+ 0.01 to + 0.19	No or negligible relationship	0.61–0.80	substantial agreement
0	No relationship	0.81–0.99	near perfect agreement
-0.01 to -0.19	No or negligible relationship	1	perfect agreement
-0.20 to -0.29	weak negative relationship		
-0.30 to -0.39	Moderate negative relationship		
-0.40 to -0.69	Strong negative relationship		
-0.70 or higher	Very strong negative relationship		

Analysis was carried out for the entire skeleton and subsequently separated according to body regions, i.e. head, upper extremity, lower extremity, body trunk (thorax, spine, pelvis) and small bones (hand and foot skeleton). In this context, “consensus” refers to all fractures that were considered proven in the overall view of the clinical and radiological examinations and, if necessary, additional MRI or other further radiological diagnostics.

## Ethics vote

The study was approved by the Ethics Committee of the University Hospital Jena (registration number 2018-1138-BO) and the performance of the additional scintigraphy was approved by the Federal Office for Radiation Protection Germany with the patient’s consent (approval number Z 5–22464/2019-110-G, valid from 03/09/2019 to 18/06/2021).

## Results

In order to include a statistically significant number of patients in the study, all patients with an ISS ≥ 9 who were admitted to hospital during the study period approved by the Federal Office for Radiation Protection had to be included.

**Fig. 1:**
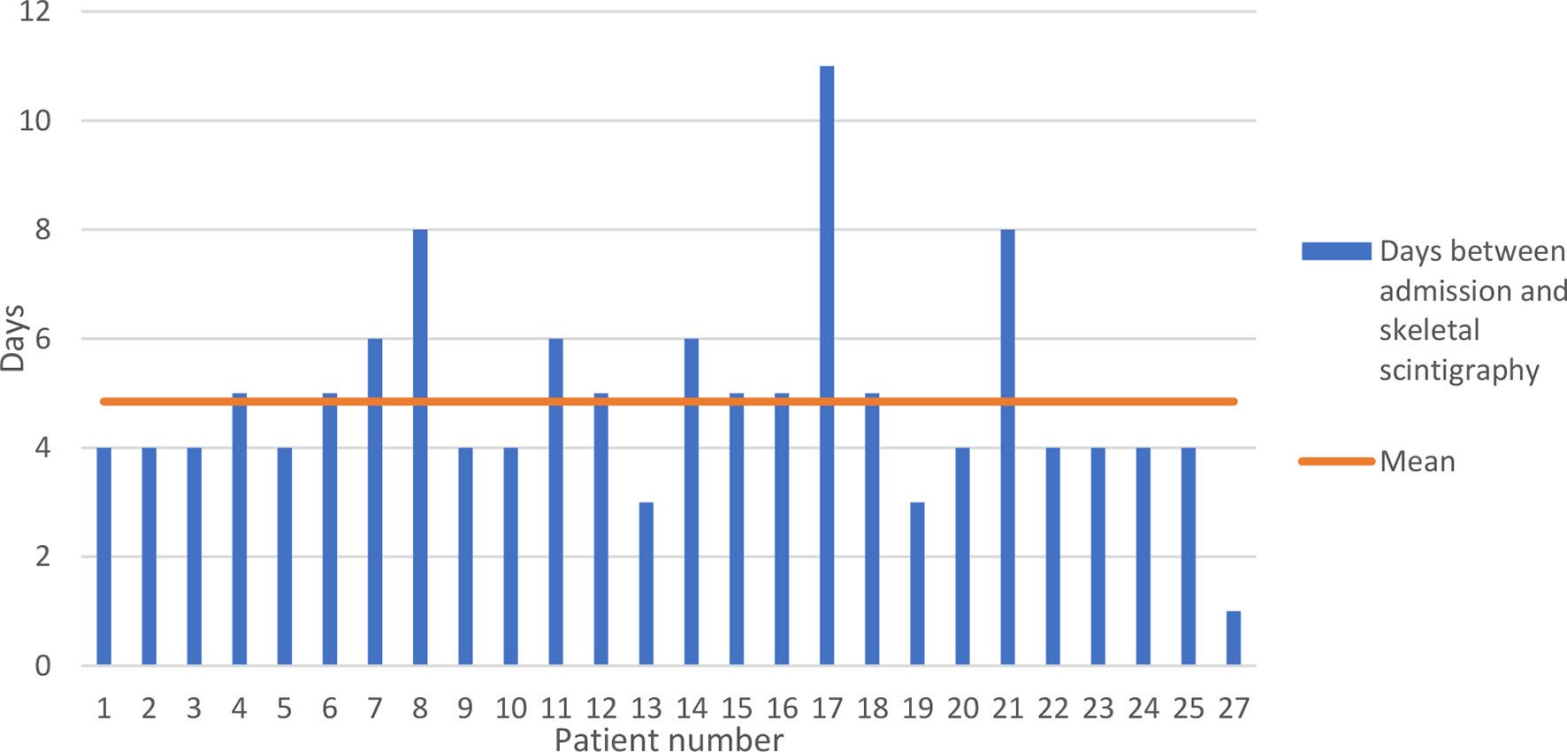
Time between admission and skeletal scintigraphy [d]

A total of 26 patients (4 female, 22 male) with a mean age of 52.9 years (range, 20 to 83 years) were included in the study. Fourteen patients had an ISS of 9 to 15 points, and 12 had an ISS of ≥ 16 points (ISS 9 to 29, mean 16.2 points). All patients were alert, oriented, and cooperative upon arrival at the emergency department. The additional physical examination as part of the tertiary survey took place on average after 4.9 days (1 to 11 days) and the skeletal scintigraphy after an average of 7.7 days (5 to 21 days) (Fig. 1).

In total, 256 fractures were detected in 26 patients by means of diagnostics (consensus). Fractures at 4164 localisations were excluded (consensus).

By means of the diagnostics in the trauma room (Table 2), i.e. clinical examination, polytrauma spiral CT and, if necessary, additional X-ray diagnostics in the trauma room, 187 fractures could be correctly identified. 69 fractures were not detected (Table 3). This corresponds to a sensitivity of 73.0%. In 5 further recognised fractures, it was found in the course of treatment that there was no fracture (3x cervical spine, 1x thoracic spine, 1x lumbar spine).

The tertiary survey (Table 4), i.e., the repeat clinical examination after an interval, correctly identified 6 additional fractures (sensitivity 75.4%) and suspected fractures in 51 regions that were not confirmed in the further course. After completion of the standard procedure (trauma room diagnostics and tertiary survey), 63 fractures were not known in seriously injured patients and, in the overall view of the available findings in these areas, there was no indication for further diagnostics.

In the skeletal scintigraphy (Table 5), which was carried out as a supplementary examination in the context of this study, 252 fractures were detected (sensitivity 98.8%). Three fractures were not detected, including 2 metacarpal fractures that were not detected in the scintigraphy due to carpal fractures and insufficient Spatial resolution. They were later detected in the supplementary diagnostics due to the suspected carpal fractures. There was also one scapular fracture: an explanation for this is probably the proximal localisation to the body trunk and the early skeletal scintigraphy in this case after only 5 days post trauma. According to experience, these regions accumulate later than, for example, distal extremities near joints in the case of injuries during scintigraphy [7]. in 25 regions, a fracture was suspected, which was not confirmed by the overall findings (Table 6). in these cases, the re-evaluation of the previous diagnostic procedures and the additional MRI tests revealed only degenerative changes.

Scintigraphy has by Far the highest sensitivity at 98.8%. Only 3 of 256 fractures were not detected. Trauma room diagnostics have a sensitivity of only 73%. Sixty-nine fractures were not detected in the trauma room. In particular, rib fractures and fractures of the hand and foot skeleton were not detected. At the same time, the emergency room diagnostic investigation has the highest specificity at 99.9%. Only five vertebral fractures were not confirmed. The specificity of the scintigraphy is somewhat lower at 99.4%. Twenty-five suspected fractures were later found to be degenerative changes. The tertiary survey with a further clinical examination only slightly increased the sensitivity. Only 6 further fractures were diagnosed. However, 51 fractures that had been suspected in the clinical examination were not confirmed. The sensitivity is therefore only 75.4% (Table 7).

**Table 3:** Statistical calculations, trauma room diagnostics (clinical examination, polytrauma spiral CT and, if necessary, additional radiological diagnostics in the trauma room)

		Consensus	
		Positiv	Negativ
**Trauma room diagnostics**	positiv	187	5
	negativ	69	4159
sensitivity	false positive	73,0%	0,1%
false negative	specificity	27,0%	99,9%
	PPV	97,4%	
	NPV	98,4%	
	prevalence	5,8%	
			p-value
	Phi	0,836	0,000
	Kappa	0,826	0,000

**Table 4:** Fractures not detected in the emergency room diagnostics. Detection using scintigraphy. *Detection using X-rays

Pat. ID	Fractures that were not recognized in the trauma room diagnostics	Qty	Consequence
1	thoracic vertebra 7 fracture thoracic vertebra 8 fracture	2	conservative conservative
2	os metatarsale 4 fracture manubrium sterni fracture	2	immobilisation conservative
3	serial rib fractures 4-11 os trapezium fracture	9	additional respiratory therapy immobilisation
4	os hamatum avulsion os trapezium avulsion metacarpale fracture 2 + 4*	2	hand was already immobilised due to thumb fracture
5	rib fracture 1 right rib fracture 1 + 2 left corpus sterni fracture	4	conservative conservative conservative
6	minimal dislocated lateral longitudinal patellar fracture	1	conservative
7	serial rib fractures 7 -11 spinosus process fracture L4	6	conservative conservative
8	serial rib fractures left 5-8 serial rib fractures right 9–11	7	additional respiratory therapy additional respiratory therapy
9	fracture of the 5. toe infraction medial tibial plateau fracture of the patella fracture T8	4	immobilisation conservative conservative conservative
10	fracture L1	1	conservative
11	infraction of the medial femoral condyle non-displaced fracture of the medial tibial plateau osteochondral avulsion on the posterior surface of the patella	3	conservative conservative conservative
12	small avulsion at the greater trochanter non-displaced scapula fracture rib fractures 9 + 10	4	conservative conservative conservative
13	infraction of the medial femoral condyle	1	conservative
17	rib fracture 6 right serial rib fractures 3–7 left	6	conservative conservative
21	infraction of the medial femoral condyle infraction of the lateral tibial plateau non-displaced fracture of the talus infraction of the distal fibula infraction at the medial malleolus serial rib fractures 3–6 right infraction proximal fibula infraction proximal Tibia bone bruise of the lateral femoral condyle manubrium sterni fracture scapula fracture	13	conservative conservative immobilisation conservative conservative conservative conservative conservative conservative conservative conservative
22	fracture L5 corpus sterni fracture	2	conservative conservative

**Table 5:** Statistical calculations, trauma room diagnostics and tertiary survey (clinical examination, polytrauma spiral CT and, if necessary, additional radiological diagnostics in the trauma room and additional clinical examination after an interval)

		Consensus	
		Positiv	Negativ
**Trauma room diagnostics** ** + ** **tertiary survey**	positiv	193	51
	negativ	63	4113
sensitivity	false positive	75,4%	1,2%
false negative	specificity	24,6%	98,8%
	PPV	79,1%	
	NPV	98,5%	
	prevalence	5,8%	
			p-value
	Phi	0,759	0,000
	Kappa	0,758	0,000

**Table 6:** Statistical calculations from skeletal scintigraphy

		Consensus	
		Positiv	Negativ
**Skeletal scintigraphy**	positiv	253	25
	Negativ	3	4139
sensitivity	false postive	98,8%	0,6%
false negative	specificity	1,2%	99,4%
	PPV	91,0%	
	NPV	99,9%	
	prevalence	5,8%	
			p-value
	Phi	0,945	0,000
	Kappa	0,944	0,000

**Table 7:** Localisation of fractures over-diagnosed in scintigraphy (joint-related degenerative changes)

Region	Qty
close to the acromioclavicular joint	3
close to the hip joint	1
close to the carpus and metacarpus	10
close to the knee joint	7
close to the ankle joint	3
ulnar shaft	1

**Table 8:** Sensitivity and specificity of the examinations for the different regions (upper and lower extremities, each without hands or feet)

Diagnostic	Sensitivity / Specificity	Total [%]	Head [%]	Upper extremity [%]	Lower extremity [%]	Trunk [%]	Bones of the hands or feet [%]
**Trauma room diagnostics**	Sensitivity	73.0	100.0	100.0	46.2	75.6	63.6
	Specificity	99.9	100.0	100.0	100.0	99.6	100.0
**Trauma room diagnostics + tertiary survey**	Sensitivity	75.4	100.0	100.0	61.5	76.2	68.2
	Specificity	98.8	95.6	99.6	99.1	98.6	98.7
**Scintigraphy**	Sensitivity	98.8	100.0	100.0	100.0	99.5	90.9
	Specificity	99.4	100.0	99.8	98.2	99.8	99.4

A subgroup analysis was performed (severely injured patients with ISS 9 to 15 vs. polytraumatized patients with ISS ≥ 16). This showed a significantly higher sensitivity in the group with ISS 9 to 15 compared to the group with ISS ≥ 16 for shock room diagnostics (66.9% vs. 80.7%) and the combination of shock room diagnostics and tertiary survey (68.3% vs. 84.2%). In case of skeletal scintigraphy, there was only a marginal difference (98.6% vs. 99.1%), with a significantly increased sensitivity of nuclear medicine diagnostics compared to standard diagnostics.

**Fig. 2:**
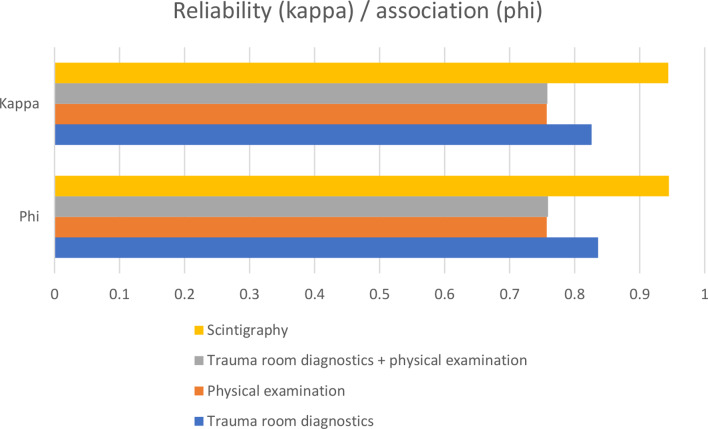
Kappa and phi for the various examinations for the whole body

The calculation of kappa and phi, as values for reliability and correlation, also shows the best results for scintigraphy (Fig. 2).

**Fig. 3:**
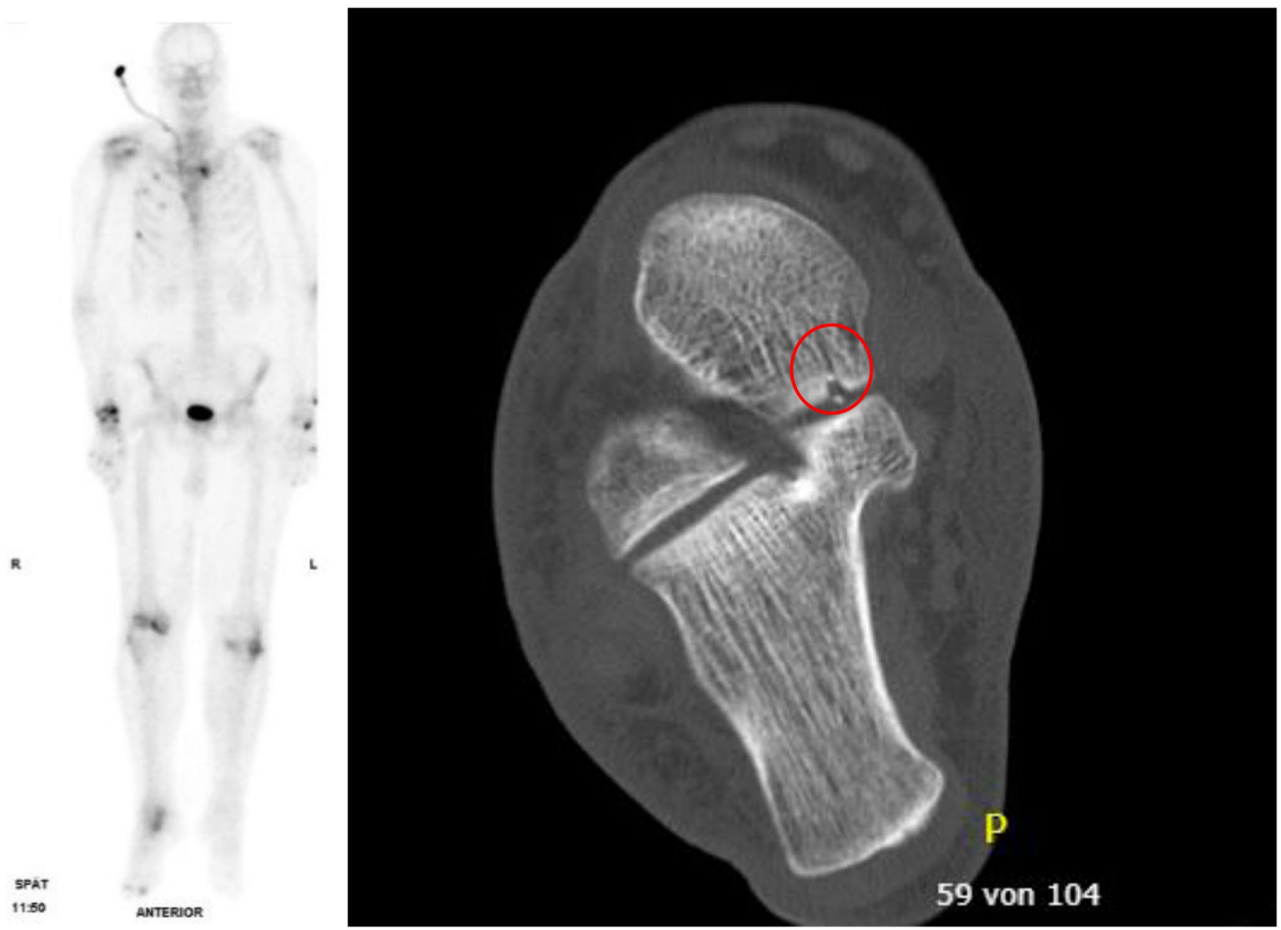
Late phase of the scintigraphy. Various rib fractures on the right and, among other features, an accumulation in the right talus can be seen well. A talus fracture (circle) that was barely visible on the CT was confirmed here

The additional scintigraphy and the resulting re-evaluation of the CT diagnostics, or extended diagnostics using CT or X-ray of certain regions and MRI diagnostics, led to the detection of a total of 60 additional fractures in 15 of 26 patients (57.7%) compared to standard diagnostics consisting of trauma room diagnostics and tertiary survey. Conservative therapy was used for 54 fractures, without additional immobilisation. In 5 fractures, affecting 4 patients, additional splints were used, for example in the case of a talus fracture that was identified only in the re-evaluation of the CT diagnostics, after which the scintigraphy showed an increased accumulation (Fig. 3). Furthermore, in one case, immobilisation could be terminated after ruling out a cervical spine injury (Table 8).

**Table 9:** Therapeutic consequences after scintigraphy and completion of additional diagnostics

	Head	Upper extremity	Trunk	Lower extremity	Bones of the hands or feet
Conservative without additional immobilisation	0	0	46	8	0
Conservative with additional immobilisation	0	0	0	1	4
Surgical	0	0	0	0	0
Termination of unnecessary immobilisation	0	0	1	0	0

## Discussion

Overlooked injuries can overshadow successful life-saving measures in the further course of treatment and may have legal consequences. In this study, additional skeletal injuries were detected in 15 of 26 patients (57.7%) by means of supplementary skeletal scintigraphy. These injuries were not detected by primary diagnostics in the trauma room and a routine third clinical examination a few days after the accident. This is a significantly higher percentage than reported in the literature, where missed injuries are reported in 0.82 to 39% of patients [2, 3, 8–10], and was possible only with the use of highly sensitive skeletal scintigraphy. It was only through this that there were indications in 63 of 256 fractures (24.6%) that fractures had been overlooked in the previous diagnostics or that further diagnostics were required. Reliable avoidance of overlooked injuries by introducing a structured “tertiary survey” with the support of an app, as reported by Moffat et al. 2019 [11], is likely to be an illusion.

Although only alert and cooperative patients were included in this study, the primary trauma room diagnostics with ATLS-compliant primary and secondary survey, including polytrauma CT, showed a sensitivity of only 73% for a patient’s bony injuries, which could be increased by only 2.4–75.4% with a tertiary survey. In particular, fractures of the ribs, avulsions of the lower extremities and of the small bones in the hands and feet were identified only in the scintigraphy. In the case of the ribs, this could be due to the axial slices of the polytrauma CT not being orthogonal to the ribs. In the future, computer algorithms such as synbo.CT Bone Reading from Siemens [12] or artificial intelligence could help in such cases [13]. In the area of the extremities, the classic CT polytrauma spiral has two problems. In this study, the upper extremities were placed on the abdomen; hence, all slices had to be calculated in order to achieve an optimal slice direction to the carpal region and, moreover, artefacts caused by the trunk could not be avoided. In the area of the lower extremity, only an initial imaging is carried out in the trauma room if there are indications of injuries below the pelvis in the primary or secondary survey, so that these regions were in some cases not imaged.

In the authors’ opinion, a supplementary skeletal scintigraphy is a safe way to detect “all” bony injuries in severely injured patients and the minimal additional radiation exposure is acceptable (Tables 9, 10). The majority of the additionally detected fractures had no therapeutic relevance in terms of additional immobilisation or surgery. However, even in this study with a relatively small number of patients, 5 fractures (7.2%) required additional immobilisation, which would not have occurred without the information provided by scintigraphy. The additional information obtained from skeletal scintigraphy could also be relevant in the long-term follow-up of patients with persistent complaints, in the context of assessments for compensation claims, accident insurance or pension applications.

### Limitations of the study

Only a relatively small number of patients could be enrolled because the use of radiation on patients in Germany is subject to an extensive approval procedure and only very few and very old preliminary studies were available to indicate a benefit for patients. The inclusion of patients had to be stopped when the approval of the Federal Office for Radiation Protection and the insurance company expired.

Unfortunately, only conscious patients could be included in the study, as there is no neurosurgery on site at St. Vincenz Hospital and patients with craniocerebral trauma had to be transferred elsewhere. Accordingly, all patients were able to provide detailed information in the trauma room and during the tertiary survey about where they had pain or restricted mobility. In intubated and mechanically ventilated patients, even more fractures would probably have been detected only during skeletal scintigraphy. This requires further study at a level I trauma center.

**Table 10:** Radiation exposure from various diagnostic procedures [14–17]

Radiological/nuclear medicine procedure		Radiation exposure
3-phase whole body skeletal scintigraphy with Tc99m/HDP		500 MBq corresponds to 2.85 mSv
Conventional x-ray	thoracic X-ray posterior-anterior	0.02 mSv
	lumbar spine	2.4 mSv
Computed tomography	skull	2 mSv
	thorax and abdomen	8 mSv
	polytrauma spiral examination	31 mSv

## Data Availability

Data available on request from the authors.

## References

[1] Enderson BL, Maull KI. Missed injuries. The trauma Surgeon’s nemesis. Surg Clin North Am. 1991;71(2):399–418. 10.1016/s0039-6109(16)45387-9. 2003258 10.1016/s0039-6109(16)45387-9

[2] Pfeifer R, Pape HC. Missed injuries in trauma patients: A literature review. Patient Saf Surg. 2008;2:20. 10.1186/1754-9493-2-20. 18721480 10.1186/1754-9493-2-20 PMC2553050

[3] Lawson CM, Daley BJ, Ormsby CB, Enderson B. Missed injuries in the era of the trauma scan. J Trauma. 2011;70(2):452-6; discussion 6–8. 10.1097/TA.0b013e3182028d71 10.1097/TA.0b013e3182028d71 21307747

[4] Kok D, Oud S, Giannakópoulos GF, Scheerder MJ, Beenen LFM, Halm JA, et al. Delayed diagnosed injuries in trauma patients after initial trauma assessment with a total-body computed tomography scan. Injury. 2024;55(5):111304. 10.1016/j.injury.2023.111304. 38171970 10.1016/j.injury.2023.111304

[5] Spitz J, Becker C, Tittel K, Weigand H. [Clinical relevance of whole body skeletal scintigraphy in multiple injury and polytrauma patients]. Unfallchirurgie. 1992;18(3):133–47. 10.1007/BF02588265. 1636218 10.1007/BF02588265

[6] Runkel M, Wenda K, Rudig L, Steinert H, Grebe P. [Detection of primarily unrecognized fractures in severely injured patients by skeletal scintigraphy]. Aktuelle Traumatol. 1993;23(5):230–4. 7901976

[7] Spitz J, Lauer I, Tittel K. [The age dependence of traumatically induced bone remodeling as studied in the bone scintigram]. Nuklearmedizin. 1991;30(5):155–60. 1800938

[8] Chen CW, Chu CM, Yu WY, Lou YT, Lin MR. Incidence rate and risk factors of missed injuries in major trauma patients. Accid Anal Prev. 2011;43(3):823–8. 10.1016/j.aap.2010.11.001. 21376872 10.1016/j.aap.2010.11.001

[9] Kalemoglu M, Demirbas S, Akin ML, Yildirim I, Kurt Y, Uluutku H, et al. Missed injuries in military patients with major trauma: original study. Mil Med. 2006;171(7):598–602. 10.7205/milmed.171.7.598. 16895123 10.7205/milmed.171.7.598

[10] Tammelin E, Handolin L, Söderlund T. Missed injuries in polytrauma patients after trauma tertiary survey in trauma intensive care unit. Scand J Surg. 2016;105(4):241–7. 10.1177/1457496915626837. 26929292 10.1177/1457496915626837

[11] Moffat B, Vogt KN, Inaba K, Demetriades D, Martin C, Malthaner R, et al. Introduction of a mobile device based tertiary survey application reduces missed injuries: A multi-center prospective study. Injury. 2019;50(11):1938–43. 10.1016/j.injury.2019.08.020. 31447214 10.1016/j.injury.2019.08.020

[12] Thomas CN, Lindquist TJ, Paull TZ, Tatro JM, Schroder LK, Cole PA. Mapping of common rib fracture patterns and the subscapular flail chest associated with operative scapula fractures. J Trauma Acute Care Surg. 2021;91(6):940–6. 10.1097/TA.0000000000003382. 34417408 10.1097/TA.0000000000003382

[13] Li N, Wu Z, Jiang C, Sun L, Li B, Guo J, et al. An automatic fresh rib fracture detection and positioning system using deep learning. Br J Radiol. 2023;96(1146):20221006. 10.1259/bjr.20221006. 36972072 10.1259/bjr.20221006 PMC10230380

[14] Oestmann J-W. Radiologie: vom fall Zur diagnose. Georg Thieme; 2005.

[15] Davies RM, Scrimshire AB, Sweetman L, Anderton MJ, Holt EM. A decision tool for whole-body CT in major trauma that safely reduces unnecessary scanning and associated radiation risks: an initial exploratory analysis. Injury. 2016;47(1):43–9. 10.1016/j.injury.2015.08.036. 26377772 10.1016/j.injury.2015.08.036

[16] Ernstberger A, Schreyer A, Schleder S, Baumer S, Angerpointer K, Diepold E et al. Computertomographie bei Polytrauma. Trauma und Berufskrankheit2017. pp. 57–63.

[17] Party RW. Making the best use of a department of clinical radiology: guidelines for Doctors. 4th ed. The Royal College of Radiologists London; 1998.

